# Visualizing Inequality in Health and Socioeconomic Wellbeing in the EU: Findings from the SHARE Survey

**DOI:** 10.3390/ijerph17217747

**Published:** 2020-10-23

**Authors:** Aurea Grané, Irene Albarrán, Roger Lumley

**Affiliations:** Statistics Department, Universidad Carlos III de Madrid, 28903 Getafe, Spain; irene.albarran@uc3m.es (I.A.); rogerlumley88@gmail.com (R.L.)

**Keywords:** ageing, clustering, dependency, long-term care, wellbeing

## Abstract

The main objective of this paper is to visualize profiles of older Europeans to better understand differing levels of dependency across Europe. Data comes from wave 6 of the Survey of Health, Ageing and Retirement in Europe (SHARE), carried out in 18 countries and representing over 124 million aged individuals in Europe. Using the information of around 30 mixed-type variables, we design four composite indices of wellbeing for each respondent: self-perception of health, physical health and nutrition, mental agility, and level of dependency. Next, by implementing the *k*-prototypes clustering algorithm, profiles are created by combining those indices with a collection of socio-economic and demographic variables about the respondents. Five profiles are established that segment the dataset into the least to the most individuals at risk of health and socio-economic wellbeing. The methodology we propose is wide enough to be extended to other surveys or disciplines.

## 1. Introduction

Ageing, dependency and the distribution of resources for long-term care are issues of vital importance in today’s modern society. Europe, in particular, has undergone a demographic shift, with birth rates declining while longevity increases. In fact, it is estimated that, by 2060, over 30% of the European population will be over 65, with 12% of the total population being over 80 [1]. This has led to an increased interest from governing institutions in understanding how and where exactly this change is happening so that policy, both at a national and European level, can be better tailored to meet the needs of this evolving situation [2].

Of particular interest is the long-term care needs of older individuals, particularly of those who have disabilities that prevent them from performing certain tasks that relate to daily life. Individuals who require such long-term care are said to be dependent. Dependency is highly complex and intersectional with many factors. These can be physical (age, gender); psychological (depression, mental health issues); cognitive (education); economic (geographical location, access to public services); social (relationships, family); etc. [3].

It is important to view dependency from a holistic approach, understanding all factors that impact on an individual’s level of dependency. Being considered dependent by the state is often the only way an individual can gain access to a variety of public services related to long-term care. However, how an individual is designated “dependent” or not is remarkably heterogeneous across Europe [4,5]. Long-term care services are essential for older people with dependency and include a range of services, including domestic help, personal care, nursing care, general health care, and services such as “meals-on-wheels” [6]. In addition, there is a large reliance on informal care, in particular in Southern European countries, which is affected by national policy [7,8].

Another commonly accepted definition of dependency is that of the Recommendation No. R(98) 9 of Council of Europe that states dependency is “such state in which people, whom for reason connected to the lack or loss of physical, mental or intellectual autonomy, require assistance and/or extensive help in order to carry out common everyday actions” (adopted by the Committee of Ministers on 18 September 1998, at the 641st meeting of the Ministers’ Deputies, https://www.coe.int/en/web/cdcj/recommendations-resolutions-guidelines). Many other definitions exist in other countries or institutions, such as that of the World Health Organization (WHO), which states that dependency relates to any restriction or lack of ability to perform an activity in the manner or within the range considered normal for a human being [9].

While definitions of who is dependent differ from country to country and institution to institution, it is without doubt that disability increases with age. Given the demographic changes discussed above, it is essential that European countries prepare for the ageing population and their increased reliance of public long-term care services. As this change is European wide, understanding who and where the most dependent populations are can inform government institution and shape how and where any policy work should be directed.

In this paper, we use the Survey of Health, Ageing and Retirement in Europe (SHARE) as a useful tool to track this phenomenon across Europe. SHARE is a multidisciplinary and cross-national panel database of micro data on health, socio-economic status and social and family networks of about 140,000 individuals aged 50 or older (around 380,000 interviews from the beginning to date). It covers 27 European countries and Israel. It was founded in 2002 and is coordinated centrally at the Munich Center for the Economics of Aging (MEA), the Max Planck Institute for Social Law and Social Policy (Munich, Germany). SHARE is harmonized with its role models and sister studies the US Health and Retirement Study (HRS) and the English Longitudinal Study of Ageing (ELSA). This model has sparked and informed exciting new survey research on ageing all over the world, e.g., Japan (JSTAR), China (CHARLS), Brazil (ELSI), South Korea (KLOSA), and India (LASI), which puts SHARE into a truly global perspective. The SHARE interview is ex-ante harmonized and all aspects of the data generation process, from sampling to translation, from fieldwork to data processing, have been conducted according to strict quality standards. As a result, SHARE has the advantage of encompassing cross-national variation in public policy, culture and history across a variety of European countries. SHARE has therefore become a major pillar of the European Research Area with over 9000 researchers registered as SHARE users. More than 1350 journal papers have been published using this data source, and over 2300 publications have cited SHARE data in their papers. Further details can be found on their website (http://www.share-project.org/).

The main contribution of this paper is to propose a procedure to visualize profiles of individuals, that is, groups of individuals with similar characteristics, using mixed-type survey data (qualitative and quantitative individual responses). In particular, we design four health and socio-economic wellbeing composite indices that are used to obtain the profiles and essential to better understand and interpret them. Next, all socio-economic and demographic variables about the respondents are combined with these indices via the k-prototypes algorithm [10] and clustered to create several profiles. The methodology proposed is wide enough to be extended to other surveys or disciplines.

This paper aims to provide a useful guide to the social and economic researchers in the visualization of profiles of individuals, that is, groups of individuals with similar characteristics, from very large mixed datasets using existing tools.

## 2. Literature Review

Today, there is still an open debate about the concept of wellbeing and how to measure it properly in individuals. This concept of wellbeing can incorporate several variables depending on the field of study (medicine, health, demography, politics, etc.). The most common characterization considers no mutually exclusive approaches, such as subjective, objective, material and relational wellbeing. See references [11,12,13,14,15] for different approaches on categorizing wellbeing. 

There are plenty of studies measuring wellbeing, either focusing on general population or on particular groups such as children, the elderly, women, minorities and others. See references [16,17] among others. Some studies of note include references [18,19], which discuss how feeling useful in life halves the risk of developing disabilities related to ADLs. Walker (2004) [20] highlights the importance of objective health and wellbeing in the quality of life of older people. It can be seen in references [21,22] that health status shows a strong relationship with quality of life, mainly during the second half of life. 

According to the worldwide study by Steptoe et al. (2015) [23], wellbeing and health are closely related to age. Thus, wellbeing seems to play a protective role in being healthy, although other factors may be considered when studying longevity. In fact, mobility restrictions and other functional disorders related to ADLs and IADLs can be considered relevant issues in wellbeing and should be considered in any study on the subject [24,25]

Cristea et al. (2000) [26] considers a major challenge for the European Union: public health expenditure is mainly focused on older, dependent people. Sustainable economic development relies on a well-balanced workforce of young and old. If an imbalance in favor of older people occurs, productivity will suffer, and older people demand more health services.

Social protection systems (also namely social welfare models), according to Eurofound (2020) [27], are intended to protect people against the risks associated with old age, illness and disability, unemployment, costs of children and housing. In particular, article 2 of the EU Treaty identifies the promotion of a high level of social protection as a key task of the EU. Social protection systems include benefits both in cash and in kind. As such, the European Pillar of Social Rights highlights access to long-term care, healthcare, old age pensions, childcare, and social housing alongside access to unemployment benefits and minimum wage policies. 

In Esping-Andersen (1990) [28], different social welfare models are distinguished (which usually involve social policies on education, health, long-term care, etc.). Specifically, this author speaks of Anglo-Saxon, Corporative, Scandinavian, and Southern-Europe models. Today, regarding long-term care, Central and Northern European countries are adhered to the “north-continental” long-term care system, which can be seen as inheritor of Corporative and Scandinavian social welfare models. This long-term care model is financed via tax and public funds with a medium-high development of private insurance sector. On the other hand, Southern European countries tend to have the “Mediterranean” long-term care system, which is financed via tax and co-payments with a very low development of the private insurance sector. Additionally, this second long-term care model has a stronger dependency on informal care by households and families [7,8].

The social characteristics of care, such as the availability of formal care, and the proportions of people receiving various types of care (formal and informal) differ greatly in European countries [6]. The increasing of life expectancy and declining fertility rates in Europe makes care for older people one of the most important issues at a policy level [29]. A big increase in the need for care and a smaller number of potential informal carers can be expected due to the rapid ageing population.

Barczyk and Kredler (2019) [7] make a comparison of the regulations of eleven public national or regional long-term care programs in Austria, Belgium, the Czech Republic, France, Germany, Italy, and Spain. The comparison is made in terms of their degree of inclusiveness with respect to vulnerable older individual’s health status. These authors provide detailed information on assessment and eligibility frameworks for long-term care programs in Europe and compare them by using the SHARE data. 

According to Hlebec et al (2019) [29], research must focus on developing instruments and methods that produce reliable measurements on health that are comparable across countries. These tools help to better understand the health problems of older adults globally, and to facilitate appropriate health and social policy responses. Additionally, developing these tools will contribute to the collection of comparable health and ageing data across countries that in turn facilitate international comparisons and learning from the experience of others [30]. Other interesting papers about long term care, health, ageing, and wellbeing include references [31,32,33,34].

This paper aims to fill a literature gap by searching for a comprehensive, cross-national overview that takes into account key issues related to objective and subjective health and long-term care and wellbeing. In particular, this paper contributes to the study of wellbeing and health combining objective and subjective variables of mixed type from the SHARE dataset in order to visualize differences between groups of Europeans.

Having an overview of the situation in Europe is relevant for the development of socioeconomic and health policies. Most countries aim to enable people to live “longer and better.” Increasing life expectancy is not, in itself, a guarantee of a better quality of life [35].

## 3. Methodology

Mixed-type data comprise both numeric and categorical features, and mixed datasets frequently occur in socio-demographic surveys as well as in other disciplines, such as economics, health, finance, marketing, etc. One of the challenging aspects of dealing with mixed-type data is to find structures and to group similar individuals for further analysis. Additionally, the availability of large and very large datasets adds complexity to this matter. 

The method that we propose is based on two stages: The first stage consists of the design of several composite indices, each of which focusses on a particular issue of interest. In the second stage, these thematic indices are combined with other descriptive variables through a clustering procedure able to cope with large datasets of mixed data. In this work, we use the *k*-prototypes clustering algorithm [10], and we illustrate the methodology through the SHARE database.

### 3.1. First Stage

In this stage, the survey information is split into two groups: descriptive variables and thematic blocks. Next, indicators are designed within each thematic block. The procedure consists of the following steps:

(1) Select the group of descriptive variables. This group does not contribute to the indices and it is left unchanged. The kind of variables to be included in this group is usually related to socio-demographic information such as gender, age, education, employment, etc.
(2) Thematic blocks: Group the rest of information in particular issues of interest yielding thematic blocks. For example, group the variables in four or five aspects to be studied.
(3) For each thematic block, construct a composite index. To do so,
  (a) Variables within thematic blocks must be encoded. Here it can be of help to review some related literature with a view on how to treat each of the pertinent variables.
  (b) Transform all the included variables into binary variables, always maintaining the same polarity. For example, assign 1 to the worst-case scenario and 0 to the best-case scenario.
    - Always maintain consistency. For categorical variables, when a scale is identical for two or more of them, the same cut-off must be designated for these variables. For example, this means that any question where the response options are “Poor”, “Fair”, “Good”, “Very good”, or “Excellent”, these variables must be transformed in the same manner using the same cut-off point.
    - For quantitative variables, thresholds such as the median or even more restrictive percentiles can be used.
  (c) Leave binary variables unchanged.
  (d) Finally, construct the thematic index by adding all binarized variables and rescale to 0–10.

This index design has several advantages: First, the researcher can use both quantitative and qualitative variables. Second, qualitative variables can be measured in different scales. Third, the transformation into binary variables serves to unify scales and helps to identify patterns of interest. Fourth, the indices are additively constructed; thus, relevant new variables can be added if necessary.

### 3.2. Second Stage

Once thematic composite indices are constructed, in the next stage we combine them with the collection of descriptive variables, clustering the individuals in groups with similar characteristics and, thus, obtaining the profiles.

Standard hierarchical clustering methods can handle data with numeric and categorical values by using Gower’s similarity coefficient [36]. For instance, this measure was used in Albarrán et al. (2015) [4], and profiles were obtained via multidimensional scaling. However, this approach may be unfeasible for large datasets. Hierarchical clustering methods such as this have a computational complexity of O(n^2^), where n is the number of observations, and with *n* > 10,000, this reinforces the unfeasibility of applying this methodology to large and very large datasets. In our application, *n* = 60,020.

A popular algorithm in unsupervised learning and clustering is the k-means clustering method [37], which has proven to be efficient for processing large data sets, although it only works on numeric data, i.e., the variables measured on a ratio scale. In Huang (1997a) [10], a combination of the k-means with the k-modes clustering algorithm by Huang (1997b) [38] is proposed, yielding to the k-prototypes clustering algorithm, with a reduction of the computational cost to O((T+1)kn), where n is the number of observations, k the number of clusters and T the number of iterations. As (k + T + 1) << *n*, we can see this is much less computationally intensive than, for instance, calculating Gower’s similarity coefficient and applying multidimensional scaling. See references [39,40] for comprehensive reviews of clustering methods for mixed data.

(1) $$d_{2}\left(x_{i},\;x_{l}\right)=\overset{p}{\underset{j=1}{\sum }}{\left(x_{ij}-x_{lj}\right)}^{2}+\gamma \overset{m}{\underset{j=p+1}{\sum }}\delta \left(x_{ij},x_{lj}\right)$$

where δ(p,q)=0 for p=q and δ(p,q)=1 for p≠q. Note that, when γ=0 clustering only depends on numeric variables. The influence of this parameter is discussed in Albarrán et al. (2015) [15], where it is recommended to select its value in accordance with the average standard deviations of numeric variables, σ; that is, the author suggests to take γ between 1/3σ and 2/3σ. See also Huang (1998) [41]. 

As with any non-hierarchical clustering approach, the number of clusters, k, must be determined before running the algorithm. A variety of techniques are available to assist in the decision of the number of clusters to be selected. However, determining the optimal number of clusters is an inherently subjective measure that depends on the goal of the analysis. In this work, we use the “elbow” method. This method consists in running the algorithm with a varying k and calculating the cost function for each run. Next, cost function values are plotted in a line-graph. At the point where there is a turning point, or “elbow”, the improvement in the cost function levels off. That is to say, the improvement by adding an additional grouping in the data is marginal, and the added complexity of understanding the profiles outweighs any benefits given.

Finally, the description of the profiles can be done by looking at the “average” individual of each profile, regarding wellbeing indicators and descriptive variables. The modal class can be used for categorical variables, and for quantitative ones, either the mean or the median may be useful.

## 4. Data and Methods

Wave 6 of the Survey of Health, Ageing and Retirement in Europe (SHARE) is a rich panel database of individuals aged 50 or over in 18 European countries and Israel. This survey took place in 2015 and asked questions ranging from the respondents’ financial situation to their self-perception of their health levels [42]. This survey is the only of its type that collects homogeneous information of this nature. This massive dataset aims to be representative; the 60,020 cases selected for this analysis include a weighting variable that scales to represent a target population of 124,313,623 aged 55 years or over individuals in Europe [43].

The data also contained many descriptive information about the respondent, such as their level of education, their marital status and others. A presentation of the descriptive variables can be found below and a description of how they were treated is in Section 4.1. To create the profiles, the survey responses were recoded into binary variables, and four indices were created. These were then used in the final clustering algorithm, along with the categorical data, to create the profiles. The process of creating the indices is described in Section 4.2, below.

Descriptive socio-economic variables included in the analysis and their possible values or categories:

- Country: 18 European countries and Israel;
- Gender: Male, Female;
- Ages: 55–60, 61–65, 66–75, 76+;
- Employment status: Employed, Not working;
- Marital status: Has no spouse, Has a spouse;
- Children: Has no children, Has one or more children;
- Education: No education, Primary, Secondary, University;
- Household in financial distress: Yes, No;
- Household receives benefits or has payments: Payments and no benefits, No benefits and no payments, Benefits and payments, Payments and no benefits.

### 4.1. Descriptive Variables

Age was split into brackets of five years, starting at 55 years. Marital status and employment status have been classed into binary responses following Albertini and Arpino (2018) [44]: either the individual has a spouse or they do not (including widowed individuals) and similarly either they are working or they are not (including retired individuals). Education has been classified into four classifications following Becchetti et al. (2015) [45]: no education, primary education, secondary education, and university education. Finally, as the focus of this paper is less on the financial aspects of ageing, the financial metrics were summarized into a combination of whether the respondent received payments from the government and whether they had out-of-pocket medical expenses. Binary variables, such as whether the respondent had children or not, were left as is. An interesting variable is financial distress, which is a binary variable (responses Yes or No) on whether the household has trouble making ends meet from month to month.

### 4.2. Index Creation

In order to cluster the data efficiently, four indices were created from 28 variables listed in Table 1. The data was grouped by variables that dealt with (1) the respondents’ self-perception of their health, (2) their physical health and nutrition, (3) their mental agility, and (4) their level of dependency. To encode these variables, papers using the SHARE dataset were reviewed with a view on how to treat each of the pertinent variables. All the included variables were transformed into binary variables. For each variable, the worst-case scenario was given the value 1, while the better scenario was given the value 0.

To maintain consistency, when a scale was identical for two or more variables, the same cut-off was designated for these variables. For example, this meant that any question where the response options were “Poor”, “Fair”, “Good”, “Very good”, or “Excellent”, these variables were transformed in the same manner using the same cut-off point. As with the descriptive variables, binary variables were left unchanged.

Once the variables had been recoded to binary values, they were summed and rescaled from 0 to 10. We start by describing which variables were used in the creation of which index. A description of the process and reasoning follows.

- **Index 1: Self-perception.** Life satisfaction; Life happiness; Self-perceived health; of health EURO depression scale; Satisfied doing no activities last year.
- **Index 2: Physical health and nutrition.** Number of chronic diseases; Number of nights spent in hospital; Living in a nursing home; Eyesight score; Hearing score; Ever smoked daily; How often eat fish, meat or poultry; How often eat vegetables; BMI; Max grip strength.
- **Index 3: Mental agility.** Self-rated reading; Self-rated writing; Score of memory test; Score of numeracy test; Score of orientation in time test; Score of words list learning test (both trials); Score of verbal fluency test.
- **Index 4: Dependency.** Global Activity Limitation Indicator (GALI); Number of mobility limitations; Number of difficulties in ADLs; Number of difficulties in IADLs; Physical inactivity.

#### 4.2.1. Index 1: Self-Perception of Health

This index included all the variables that related to what an individual thought about their health rather than the physical reality; this is quite a subjective measure and captures some aspects of subjective wellbeing. Questions in this section were more around how satisfied the respondent was with their life, whether they were feeling depression, etc. Havari and Peracchi (2014) [46] analyzed the SHARE dataset and recoded many variables which we have also investigated in this paper. Their focus on self-perceived health was important for the construction of this particular index. Perhaps most importantly, they coded a 0 if the respondent answered “Good”, “Very good”, or “Excellent” in the self-perceived health question and a 1 for those who answered “Fair” or “Poor.” This is supported by Côté-Sergent et al. (2018) [47] who follow the same levels for recoding. This convention was followed in all variables that used the same scale. Both Havari and Peracchi (2014) [46] and Hank (2011) [48] classified a respondent with a score of 4 or less on the EURO depression scale as a 0, else a 1.

Similarly, for the life happiness variable, if the respondent was “often” happy, they were given a value of 0, else a 1. Both life satisfaction and the “Satisfied doing no activities last year” questions were on a scale of 0 to 10, the cut off was determined to be 6 or less given a 1, else a 0.

#### 4.2.2. Index 2: Physical Health and Nutrition

This index was created to capture the risk of suffering or developing serious health problems. The SHARE dataset also contains many questions about the physical health of respondents, such as BMI, nutrition, chronic conditions and others. If an individual had 2 or more chronic condition, they were given a value of 1. This convention is followed in Havari and Peracchi (2014) [46], Côté-Sergent et al. (2018) [47] and Nie and Sousa-Poza (2016) [49]. For the diet related metrics, [49] designated a 0 for individuals who responded “Less than once a week” to any of the categories. This has been applied to the “How often do you eat vegetables?” or the “How often do you eat meat, fish, and poultry?” questions, which were included in the database. Abeliansky and Strulik (2017) [50] had a unique approach to the BMI and grip strength variables. They combined these variables to create a “fragility” variable that is dependent on this combination of variables as well as the gender of the respondent. Those who were deemed “fragile” were assigned 1. Finally, if an individual had spent at least one night in hospital in the previous year, they were encoded with a 1 [51].

#### 4.2.3. Index 3: Mental Agility

This index was created to capture the mental acuteness of the respondents and is related to cognitive functions. The SHARE survey contained certain tests around numeracy, orientation and linguistic fluency; these variables were included in this index. Both the numeracy test and orientation tests were scaled on a 0–5 scale. This was encoded to a 1 if the respondent scored a 1, 2, or 3 [46]. For the fluency and word list tests, Hank (2011) [48] combined the scores of these three metrics, then used the median as the cut-off to convert this to a binary variable.

#### 4.2.4. Index 4: Dependency

As stated in Section 1, dependency is measured and defined in surprisingly heterogeneous ways across Europe. Thus, this index has combined a variety of these methods to standardize and ensure comparability across individuals. For all the non-binary variables included in this index, an individual was given a value of 1 if they reported suffering from at least one of the corresponding conditions. Many papers follow this convention for ADL and IADL, and this was extended to number of mobility limitations [5,52].

### 4.3. Profile Construction

Once the health and wellbeing indices are constructed, the next stage of the procedure is to combine them with a collection of descriptive variables, which contain the socio-economic and demographic information, clustering the individuals in groups with similar characteristics and, thus, obtaining the profiles. To do so, we used the k-prototypes clustering algorithm. In particular, we used the k-prototypes clustering algorithm included in the k-modes Phyton package (GitHub, San Francisco, CA, USA) https://github.com/nicodv/kmodes/blob/master/kmodes/kprototypes.py.

**Figure A1:**
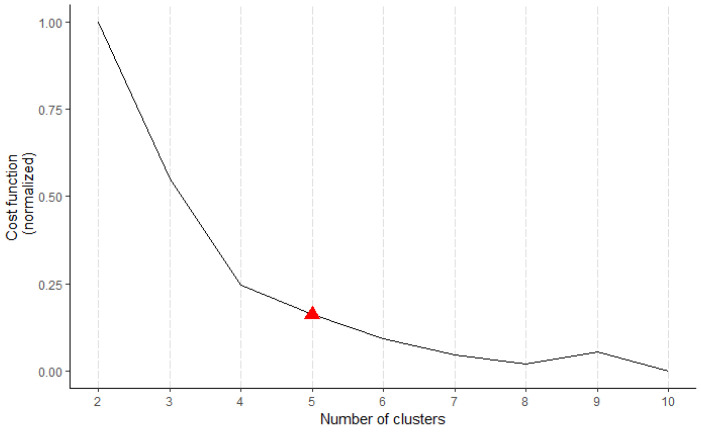
Cost function for the k-prototypes algorithm.

To select the number of clusters we run the algorithm for k = 2, 3, …, 10. The cost function showed and “elbow” around k = 4 or k = 5 (see Figure A1 in the Appendix A). Having both 4 or 5 profiles was investigated, and k = 5 was chosen, as k = 4 led to too broad results.

## 5. Results

### 5.1. Description of Profiles and Findings

**Figure 1:**
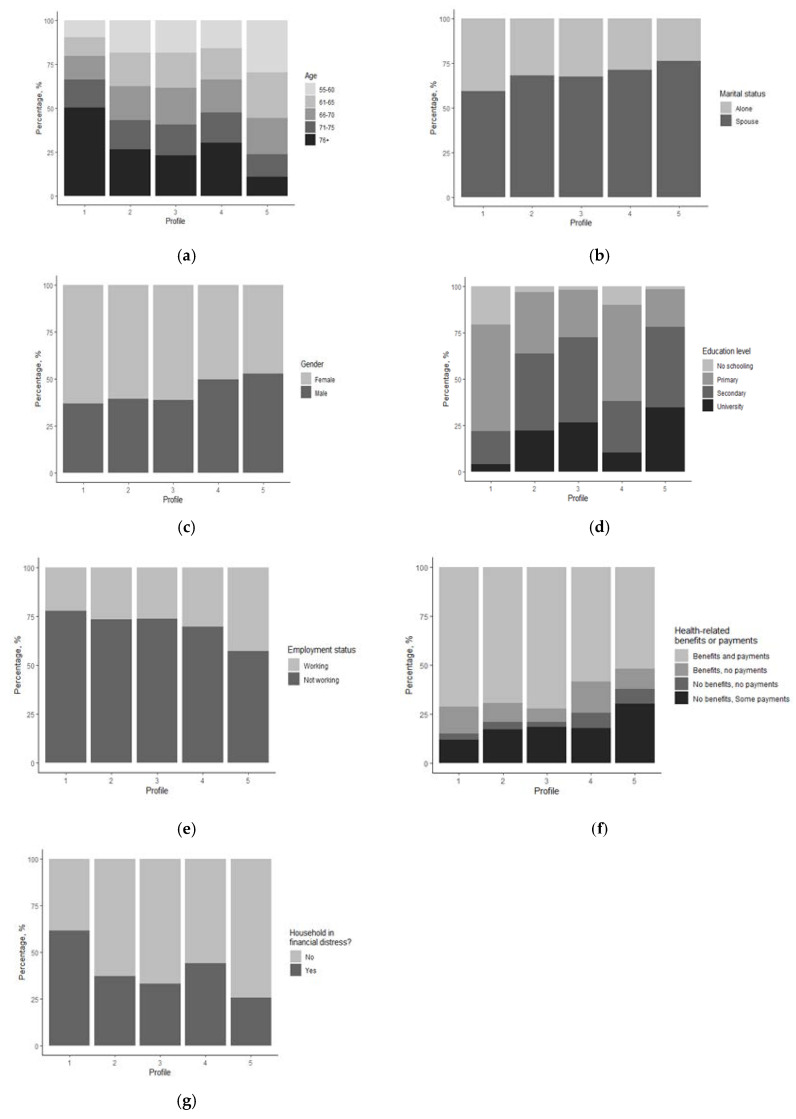
Distribution of the descriptive variables by profile. (**a**) Age; (**b**) Marital status; (**c**) Gender; (**d**) Education; (**e**) Employment status; (**f**) Benefits and payments; (**g**) Household in financial distress

With the clusters created, a profile of the average member of each cluster can be made. To do so, the profiles have been ranked by taking the mean of the mean values for each index. The profiles with higher mean values in the indices can be said to be more disadvantaged than those with lower index scores. Figure 1 in the Appendix A show the distribution of each profile for the categorical variables used in the k-prototypes clustering algorithm with k = 5. A broad summary of each profile follows.

**Figure 2:**
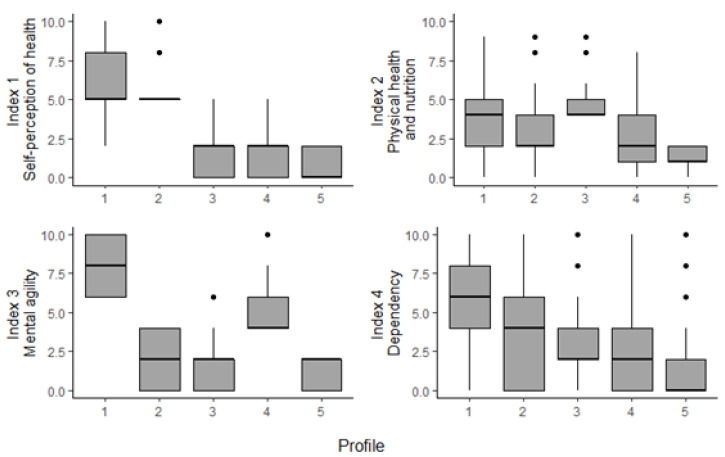
Boxplot distribution of indices by profile.

Using Figure 1 and Figure 2 and the summary of statistics of each profile included in the Appendix A (see Table A1), the following broad profiles were made.

- Profile 1: Highest social vulnerability, lowest levels of health and socioeconomic wellbeing. Urgent need of social assistance. It is composed by 15.96% of the target population. Main characteristics: female; 76 years old or older; low education (primary or none); not working; likely lives alone; suffers from multiple limitations in ADL or IDAL; health-related payments or benefits; in financial distress; very negative self-perception of health; lack of autonomy; major difficulties in cognitive functions, with risk of suffering/developing serious health conditions.
- Profile 2: Medium-high social vulnerability. It is composed by 22.59% of the target population. Main characteristics: female; equally likely to belong to any age bracket; primary educated; not working; lives with a partner; few limitations in ADL or IADL; health-related payments or benefits; negative self-perception of health; lack of autonomy; with risk of suffering/developing serious health conditions.
- Profile 3: Medium-low social vulnerability. It is composed by 10.69% of the target population. Main characteristics: female; equally likely to belong to any age bracket; secondary educated; not working; lives with a partner; few limitations in ADL or IADL; health-related payments or benefits; with some negative aspects on self-perception of health; shows the highest score of suffering/developing serious health problems.
- Profile 4: High social vulnerability, low levels of health and socioeconomic wellbeing. Urgent need of social assistance. It is composed by 23.18% of the target population. Main characteristics: male; 70 years or older; primary educated; not working; lives with a partner; some limitations in ADL or IADL; health-related payments or benefits; likely in financial distress; shows the highest score of difficulties in cognitive functions; with risk of suffering/developing serious health problems.
- Profile 5: Low risk of social vulnerability, reasonable levels of health and socioeconomic wellbeing. Least need of social assistance. It is composed by 27.58% of the target population. Main characteristics: male; younger (55–65 years old); secondary or university educated; likely still working; lives with a partner; very unlikely to have limitations in ADL or IADL; no health-related benefits, some payments; shows the lowest scores in all wellbeing indices.

From Figure 1, it can be seen that there are three female profiles and two male profiles. This is the modal class for gender for these profiles; it is not to say that every member of these profiles is female or male.

From Figure 2, it can be observed that there is quite a difference between Profile 1 and the other profiles across index 1, index 3, and index 4. Index 2 is more balanced, with the mean value of this index differing only slightly across the profiles. The female profiles rank much worse in index 1, relating to mental health issues. While women are not more likely to have more mental health issues, they are more likely to be affected by depression and anxiety, in particular as they age [53].

Index 4, which relates to dependency, shows the highest variance both within each profile and between the profiles. In fact, all profiles have individuals scoring 0 and others scoring 10 for this index. When investigating deeper into the base dataset, it can be observed that on average members of Profile 1 suffer 3 times more limitations in ADL and IADL than the sample mean, while members of Profile 5 suffer only 0.1 times the sample mean limitations. See the Appendix A for all profile results.

Profile 1 and Profile 4 are the worst off for each gender (female and male respectively), although Profile 4 is still better off than Profile 1. This is supported by the literature [54,55,56] which acknowledges the well-researched fact that women both live longer and with a poorer quality of life. These profiles also have a higher share of individuals aged 76 years or more old. For the constructed indices, they both perform badly in index 3, which relates to mental agility.

Profile 5 is the least disadvantaged profile and is heavily skewed towards younger individuals; over 50% of the individuals in this profile are aged between 55–65, while those over 76 make up less than 10% of this profile. They are the most male heavy group and also the most likely to still be working. Continuing to work in later life has been correlated to positive health outcome [2]. Across all the health indices, they score low, indicating good health. These individuals are in the least need for current social assistance.

Education is often used as a proxy for socio-economic status and its impact on later-life health outcomes is well researched [45,57,58,59]. This education disparity can be seen in the profiles. Over 75% of respondents in Profile 1 either have zero education or only primary education; for Profile 4, this number sits around 50%. Conversely, Profiles 2, 3, and 5 have barely any individuals with no education and similar levels of university level educated individuals (circa 25%).

### 5.2. Profiles across the EU

**Figure 3:**
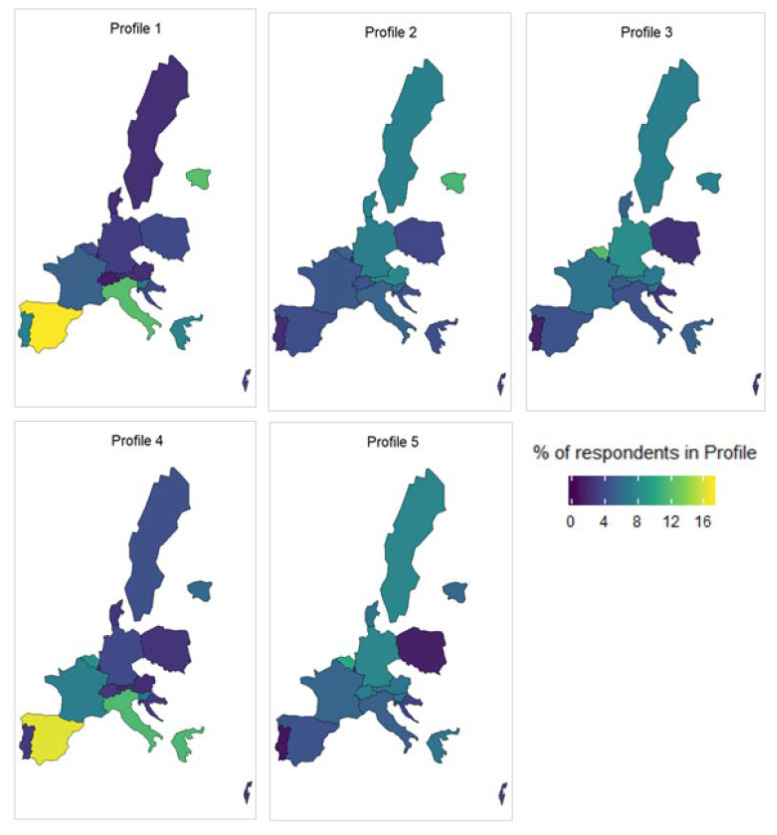
Geographical distribution of profiles 1 to 5.

The country of residence of the respondent was not included in the clustering algorithm and had no impact on the formation of the clusters. It is therefore interesting to see how the profiles are distributed across the continent and gain insight into how areas of need. This is shown in Figure 3.

One thing that is immediately of note is the distribution of the most disadvantaged profiles: Profile 1 and Profile 4. There is an over-representation of these profiles in the Southern European countries (Portugal, Spain, Italy, and Greece). As mentioned above, there is a strong relationship between education and health outcomes. This is also influenced strongly by geography [47,57]. Namely, having lower education in the Southern and Western European countries has a more negative impact on your health and long-term care needs than similarly educated individuals in the Northern European countries. This is reinforced by the findings here.

Profile 2 and Profile 3 are quite similar, with a few small differences in the distribution of their categorical variables. Where they differ more is in their geographical distribution. We can see that Profile 2 is more frequently found in the Northern and Eastern European countries (Estonia, Denmark, Poland, etc.), while Profile 3 is more prevalent around the Central European countries (Germany, Belgium, etc.). They also differ in constructed indices, with Profile 2 being slightly worse off in the majority of the four indices. In particular, Profile 2 ranks much worse in index 1, which contains variables related to depression and mental health.

## 6. Discussion

### 6.1. Key Messages and Implications

In the modern European context, it is more and more important to understand the demographic shift that has been happening for the past decades. Not only will this impact the makeup of European society, it will also present challenges for policy at a European and national level. Of particular importance are older, dependent individuals. In an attempt to homogenize the classification of these individuals, five profiles were created using the respondents to the Survey of Health, Ageing, and Retirement in Europe survey data.

To create these profiles, a clustering algorithm useful for mixed datasets, known as the k-prototypes algorithm, was implemented on the large dataset available. These profiles represent broad groupings of older Europeans and the similar struggles they face in day-to-day living.

Two of the profiles, Profiles 1 and 4, can be said to be the most disadvantaged females and males (respectively) across Europe. These individuals are older, less educated, much more likely to be dependent or have issues with ADL or IADL, and are an important subsection of society when considering policy regarding aged individuals. Interestingly, they have a similar geographic distribution, with an over-representation in Southern Europe. In fact, the well-documented Northern to Southern Europe health gradient has been observed in the distribution of the profiles. The better off profiles, such as Profiles 3 and 5, are more likely to be found in the Northern and Central European countries. Analogous patterns were found by Pappadà (2010) [60] when studying the social welfare systems in the EU from ESSPROS Eurostat dataset. A plausible explanation may be found in the diversity of social protection systems for long-term care operating in the EU.

Similar findings were pointed out by several authors. In Muir (2017) [61], it is concluded that the number of people who need help in the course of their daily lives is similar in most countries, but whether and how that help is provided varies. Although the probability of someone needing long-term care increases with age, the cross-country variation in long-term care is not in general driven by demographics. For example, the Spanish population is much older than that of the Netherlands, yet far fewer people in Spain receive long-term care. Nor is it driven by disability rates, which are thought to be similar in most OECD countries [62].

According to the Social Protection Investment in Long-Term Care (SPRINT HORIZON 2020) project, social investments mean policies designed to strengthen people’s abilities and capacities and support them to participate fully in employment and social life. The key policy areas included are education, quality childcare, medical care (including long-term care), training, job search assistance, and rehabilitation [63]. In Glanz and Fernández (2018) [64], the authors consider that the social investment approach has considerable potential to allow decision makers to strengthen long-term care systems across the EU and, therefore, help address the challenge of population ageing.

Thus, after more than thirty years of belonging to the EU wellbeing inequalities still persist in the Southern European Countries. Indeed, going further, it seems that SDG-3 of United Nations 2030 Agenda (to ensure healthy lives and promote wellbeing for all at all ages) is hardly fulfilled in these developed countries.

Undoubtedly, long-term care is expensive, and its cost varies widely between countries, being always high in relation to typical income, which means that long-term care is often unaffordable in the absence of social protection. Thus, countries should strengthen social protection systems to ensure that long-term care is not only available to the relatively wealthy [61]. A reasonable starting point maybe the proposal by Colombo et al. (2011) [65], who propose to promote healthy ageing and prevention, given that one of the most obvious ways to reduce costs in long-term care systems is to reduce potential dependence in later life by promoting health throughout life. Nevertheless, much more should be done to guarantee long-term care services across the EU.

### 6.2. Strengths, Limitations, and Future Research

In this paper, we provide a useful guide to the social and economic researchers in the visualization of profiles of individuals, that is, groups of individuals with similar characteristics, from large and very large mixed datasets by using existing tools. 

Our method is based in two stages: the first one consists in the design of several composite indices, each of which focusses on a particular issue of interest, yielding to thematic indicators. This index design has several advantages, namely the researcher can use both quantitative and qualitative variables, qualitative variables can be measured in different scales, binarization is used to track events of interest and finally their additivity allows to add relevant new variables if necessary. In the second stage, thematic indicators are combined with other descriptive variables, like socio-demographic information, through a clustering procedure able to cope with large datasets of mixed data. As a result, individuals are clustered in groups with similar characteristics and, thus, profiles are obtained. Finally, profiles can be described by looking at the “average” individual within profiles, regarding wellbeing indicators and descriptive variables.

The methodology we propose is wide enough to be extended to other surveys or disciplines.

Necessarily, the statistical analysis presented in this paper is limited to the variables available in SHARE wave 6 survey data. For instance, excessive alcohol drinking and/or drug use may have an important impact on the inequality of health in the elderly. However, this information was not available in wave 6.

An interesting area for future research would be extending and formalizing the work of Raman et al. (2011) [66]. In their paper, the authors looked into applying fuzzy clustering techniques to non-numeric data, specifically the *k*-modes algorithm. Through adapting the fuzzy centroids methodology, where an observation can belong to more than one cluster, computational performance is improved without much loss in specificity of results. Extending the fuzzy methodology to the *k*-prototypes algorithm through combining the fuzzy *k*-means and fuzzy *k*-modes algorithms, as done in Huang (1997a) [10], could lead to an improvement in the computational time.

## Figures and Tables

**Table 1:** Variables included in the analysis and their possible values or categories.

Description	Values/Categories	Description	Values/Categories
Life satisfaction	Scale, 0–10	Number of chronic diseases	From 0 to 13
Satisfied doing no activities last year?	Scale, 0–10	Number of nights spent in hospital in the past year	From 0 to 365
Self-perceived health	Poor, Fair, Good, Very Good, Excellent	Living in a nursing home	Yes, No
Life happiness	Never, Rarely, Sometimes, Often	Eyesight score (based on test)	Poor, Fair, Good, Very Good, Excellent
EURO depression scale	Scale, 0–12	Hearing score (based on test)	Poor, Fair, Good, Very Good, Excellent
Global Activity Limitation Indicator (GALI)	Limited, Not limited	Ever smoked cigarettes daily	Yes, No
Number of mobility limitations	From 0 to 10	How often consume meat, fish or poultry	Less than once a week, Once a week, Twice a week, 3–6 times a week, Every day
Number of difficulties in activities of daily living (ADL)	From 0 to 6	How often consume vegetables	Less than once a week, Once a week, Twice a week, 3–6 times a week, Every day
Number of difficulties in in instrumental activities of daily living (IADL)	From 0 to 9	BMI	From 12.5 to 98.6
Physical inactivity	Yes, No	Grip strength	From 1 to 92
Self-rated reading skills	Poor, Fair, Good, Very Good, Excellent	Self-rated writing skills	Poor, Fair, Good, Very Good, Excellent
Score of memory test	Poor, Fair, Good, Very Good, Excellent	Score of numeracy test	scale, 0–5
Score of verbal fluency test	From 0 to 97	Score of orientation in time test	scale, 0–5
Score of words list learning test—trial 1	From 0 to 10	Score of words list learning test—trial 2	From 0 to 10

## Appendix A

Summary statistics of the variables used to create the profiles, per profile. For categorical variables, the mode is supplied, as well as the proportion of that class in the cluster. For numeric variables (i.e., the indices), both the mean and median are supplied.

**Table A1:** Summary statistics of the variables used to create the profiles, per profile.

Cluster	Count	% of Total	Age	Age Prop.	Gender	Gender Prop.	Job Status	Job Status Prop.
1	19,841,335	15.96%	76+	50%	Female	63%	Not working	78%
2	28,081,489	22.59%	76+	26%	Female	61%	Not working	73%
3	13,289,581	10.69%	76+	23%	Female	61%	Not working	74%
4	28,820,451	23.18%	76+	30%	Male	50%	Not working	70%
5	34,280,767	27.58%	55-60	30%	Male	53%	Not working	57%
**Cluster**	**Household in financial distress?**	**Financial distress prop.**	**Marital status**	**Marital status prop.**	**Education level**	**Education level prop.**	**Payments or benefits?**	**Payments or benefits prop.**
1	Yes	62%	Spouse	59%	Primary	57%	B & P	71%
2	No	63%	Spouse	68%	Secondary	42%	B & P	69%
3	No	67%	Spouse	68%	Secondary	46%	B & P	72%
4	No	56%	Spouse	71%	Primary	52%	B & P	59%
5	No	74%	Spouse	76%	Secondary	43%	B & P	52%
**Cluster**	**Index 1 mean**	**Index 1 median**	**Index 2 mean**	**Index 2 median**	**Index 3 mean**	**Index 3 median**	**Index 4 mean**	**Index 4 median**
1	5.56	5	3.77	4	7.85	8	5.55	6
2	5.73	5	2.87	2	2.22	2	3.52	4
3	1.27	2	4.43	4	1.6	2	3.08	2
4	1.05	2	2.24	2	5.18	4	2.39	2
5	0.86	0	1.31	1	1.02	2	1.3	0
**Cluster**	**Average ADL limitations**		**Proportion to global average ADL limitations**		**Average IADL limitations**		**Proportion to global average IADL limitations**	
1	0.87		3.2		1.88		3.4	
2	0.33		1.2		0.61		1.1	
3	0.20		0.7		0.41		0.7	
4	0.17		0.6		0.38		0.7	
5	0.04		0.1		0.07		0.1	

B & P = Both benefits and payments.
